# The ChEMBL Database in 2023: a drug discovery platform spanning multiple bioactivity data types and time periods

**DOI:** 10.1093/nar/gkad1004

**Published:** 2023-11-02

**Authors:** Barbara Zdrazil, Eloy Felix, Fiona Hunter, Emma J Manners, James Blackshaw, Sybilla Corbett, Marleen de Veij, Harris Ioannidis, David Mendez Lopez, Juan F Mosquera, Maria Paula Magarinos, Nicolas Bosc, Ricardo Arcila, Tevfik Kizilören, Anna Gaulton, A Patrícia Bento, Melissa F Adasme, Peter Monecke, Gregory A Landrum, Andrew R Leach

**Affiliations:** European Molecular Biology Laboratory, European Bioinformatics Institute (EMBL-EBI), Wellcome Genome Campus, Hinxton, Cambridgeshire CB10 1SD, UK; European Molecular Biology Laboratory, European Bioinformatics Institute (EMBL-EBI), Wellcome Genome Campus, Hinxton, Cambridgeshire CB10 1SD, UK; European Molecular Biology Laboratory, European Bioinformatics Institute (EMBL-EBI), Wellcome Genome Campus, Hinxton, Cambridgeshire CB10 1SD, UK; European Molecular Biology Laboratory, European Bioinformatics Institute (EMBL-EBI), Wellcome Genome Campus, Hinxton, Cambridgeshire CB10 1SD, UK; European Molecular Biology Laboratory, European Bioinformatics Institute (EMBL-EBI), Wellcome Genome Campus, Hinxton, Cambridgeshire CB10 1SD, UK; European Molecular Biology Laboratory, European Bioinformatics Institute (EMBL-EBI), Wellcome Genome Campus, Hinxton, Cambridgeshire CB10 1SD, UK; European Molecular Biology Laboratory, European Bioinformatics Institute (EMBL-EBI), Wellcome Genome Campus, Hinxton, Cambridgeshire CB10 1SD, UK; European Molecular Biology Laboratory, European Bioinformatics Institute (EMBL-EBI), Wellcome Genome Campus, Hinxton, Cambridgeshire CB10 1SD, UK; European Molecular Biology Laboratory, European Bioinformatics Institute (EMBL-EBI), Wellcome Genome Campus, Hinxton, Cambridgeshire CB10 1SD, UK; European Molecular Biology Laboratory, European Bioinformatics Institute (EMBL-EBI), Wellcome Genome Campus, Hinxton, Cambridgeshire CB10 1SD, UK; European Molecular Biology Laboratory, European Bioinformatics Institute (EMBL-EBI), Wellcome Genome Campus, Hinxton, Cambridgeshire CB10 1SD, UK; European Molecular Biology Laboratory, European Bioinformatics Institute (EMBL-EBI), Wellcome Genome Campus, Hinxton, Cambridgeshire CB10 1SD, UK; European Molecular Biology Laboratory, European Bioinformatics Institute (EMBL-EBI), Wellcome Genome Campus, Hinxton, Cambridgeshire CB10 1SD, UK; European Molecular Biology Laboratory, European Bioinformatics Institute (EMBL-EBI), Wellcome Genome Campus, Hinxton, Cambridgeshire CB10 1SD, UK; European Molecular Biology Laboratory, European Bioinformatics Institute (EMBL-EBI), Wellcome Genome Campus, Hinxton, Cambridgeshire CB10 1SD, UK; European Molecular Biology Laboratory, European Bioinformatics Institute (EMBL-EBI), Wellcome Genome Campus, Hinxton, Cambridgeshire CB10 1SD, UK; European Molecular Biology Laboratory, European Bioinformatics Institute (EMBL-EBI), Wellcome Genome Campus, Hinxton, Cambridgeshire CB10 1SD, UK; Sanofi, R&D, Preclinical Safety, Industriepark Höchst, 65926 Frankfurt am Main, Germany; Department of Chemistry and Applied Biosciences, ETH Zürich, 8093 Zürich, Switzerland; European Molecular Biology Laboratory, European Bioinformatics Institute (EMBL-EBI), Wellcome Genome Campus, Hinxton, Cambridgeshire CB10 1SD, UK

## Abstract

ChEMBL (https://www.ebi.ac.uk/chembl/) is a manually curated, high-quality, large-scale, open, FAIR and Global Core Biodata Resource of bioactive molecules with drug-like properties, previously described in the 2012, 2014, 2017 and 2019 Nucleic Acids Research Database Issues. Since its introduction in 2009, ChEMBL’s content has changed dramatically in size and diversity of data types. Through incorporation of multiple new datasets from depositors since the 2019 update, ChEMBL now contains slightly more bioactivity data from deposited data vs data extracted from literature. In collaboration with the EUbOPEN consortium, chemical probe data is now regularly deposited into ChEMBL. Release 27 made curated data available for compounds screened for potential anti-SARS-CoV-2 activity from several large-scale drug repurposing screens. In addition, new patent bioactivity data have been added to the latest ChEMBL releases, and various new features have been incorporated, including a Natural Product likeness score, updated flags for Natural Products, a new flag for Chemical Probes, and the initial annotation of the action type for ∼270 000 bioactivity measurements.

## Graphical Abstract

**Graphical Abstract:**
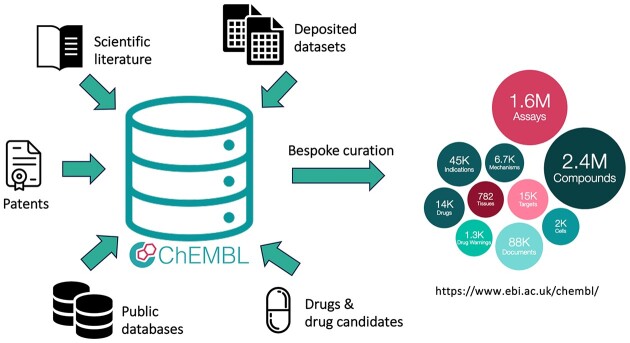

## Introduction

Since ChEMBL’s first release in 2009, scientists working in multiple sectors, including academia, not-for-profit institutes, charities, biotech companies and large global organisations have been able to access large amounts of high-quality, curated data on bioactive molecules from the medicinal chemistry literature.

To date there have been 33 separate major releases of ChEMBL, during which time the database has grown significantly in both scope and scale. A recent independent impact report, derived from a detailed survey of >4000 users, ranked ChEMBL 5th in EMBL-EBI’s most used resources, behind only the much larger and long-established gene and protein sequence databases (https://www.embl.org/documents/wp-content/uploads/2021/10/EMBL-EBI-impact-report-2021.pdf). ChEMBL is an ELIXIR core data resource (1) and in December 2022 it was included in the first list of Global Core Biodata Resources (GCBRs)—a collection of data resources recognised as critical to life science and biomedical research worldwide (https://globalbiodata.org/what-we-do/global-core-biodata-resources/list-of-current-global-core-biodata-resources/).

Critically, ChEMBL enables the scientific community to answer important science questions, including many that are health related. Recent examples from our own published work include a large-scale assessment of drug and ligand physicochemical properties and ligand efficiencies (2); the identification of drug repurposing opportunities for COVID-19 (3,4) and heart failure (5); an approach to identify targets amenable to protein degradation—‘the PROTACtable genome’ (6); the exploration of selectivity profiles for ligands of proteins commonly expressed at pharmacological barriers (7); the analysis of scaffold and target trends over time (8,9); and *in silico* target prediction models based on conformal prediction(10). There are many practical applications of ChEMBL, including the identification of tool compounds for potential therapeutic targets (11); novelty evaluation of active molecules or chemotypes (12); the creation of chemogenomic sets for phenotypic screening (13) and the identification of an active compound's potential targets and off-targets (14). Another major area where ChEMBL delivers significant impact is in data science and in the development, validation and application of AI, machine learning and other *in silico* methods (15–19).

In this 2023 ChEMBL database update, we report on new data sources, features and functionalities, as well as updates to the CHEMBL web interface. ChEMBL’s data content is ever more diverse and includes a broader coverage of target space and drugs/drug candidates for new modalities of treatment. Data deposited directly into ChEMBL represent a significant and growing part of the database which we are addressing by improved documentation for data deposition and close interaction with depositors. To facilitate data access and usage, ChEMBL’s training material has recently been revised including re-organised FAQ’s and novel open-access webinars.

## Current data content

The core content of the ChEMBL database is published bioactivity data, from a set of seven Medicinal Chemistry journals: Journal of Medicinal Chemistry, Bioorganic & Medicinal Chemistry Letters, European Journal of Medicinal Chemistry, Bioorganic & Medicinal Chemistry, Journal of Natural Products, ACS Medicinal Chemistry Letters and MedChemComm. For every paper in these journals all bioactivity measurements are regularly extracted and curated. However, the data in ChEMBL comes from ∼230 different journals spanning a broad variety of different biomedical disciplines. Journals other than the core set have less consistent coverage as data was extracted only if they were deemed interesting as part of a specific project or collaboration. Table 1 shows the 20 top journals in terms of the numbers of extracted documents, illustrating the variety of biomedical disciplines and communities covered by the data in ChEMBL, ranging from, e.g. medicinal chemistry and drug discovery to food chemistry, crop science, environmental science, and biotechnology.

**Table 1.** List of 20 top journals covered by ChEMBL 33 by number of unique documents

Journal	Number of documents	Number of assays	Number of bioactivities
J Med Chem	24 505	569 146	2 848 595
Bioorg Med Chem Lett	23 763	291 472	1 743 896
Eur J Med Chem	9410	246 065	1 460 323
Bioorg Med Chem	8873	147 680	909 607
J Nat Prod	8410	77 131	302 822
ACS Med Chem Lett	2852	52 667	216 528
Antimicrob Agents Chemother	2127	67 780	197 574
Medchemcomm	1370	25 781	133 238
Med Chem Res	1309	15 313	135 562
J Agric Food Chem	422	7232	44 962
Drug Metab Dispos	320	15 240	32 501
J Pestic Sci	245	3875	29 462
RSC Med Chem	238	4847	21 761
J Biol Chem	175	3417	6870
Nat Chem Biol	170	6553	21 679
Crop Prot	129	4984	7926
Pest Manag Sci	126	3395	9581
Proc Natl Acad Sci U S A	107	4204	44 716
J Pharmacol Exp Ther	99	660	1569
Biosci Biotechnol Biochem	64	740	6213

The numbers of assays and bioactivities associated with each Journal name are also given. Journal abbreviations are used according to NLM standards.

The current data content from literature in ChEMBL 33 spans from 1974 until 2022 demonstrating the huge value of data in ChEMBL for analyses that require a time dimension, such as time series analyses, trend analyses (8,9) or machine learning approaches that require a time split of datasets for validation purposes (20).

**Figure 1.**
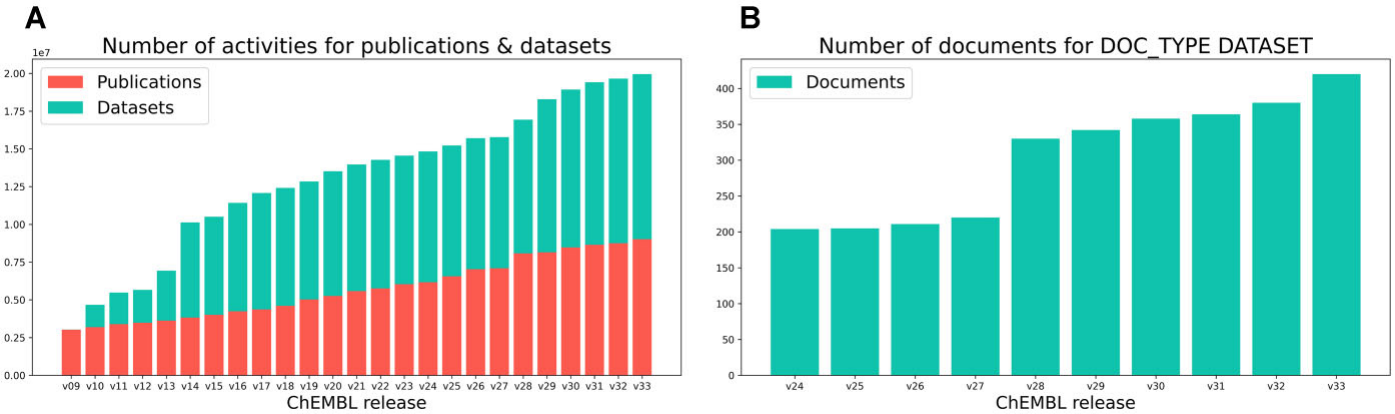
(**A**) Graph on the left shows the distribution of bioactivities in ChEMBL from different document types (‘PUBLICATION’, ‘DATASET’) over time (release v09–v33). Document types ‘PATENT’ and ‘BOOK’ have not been included in this graph since these are only assigned to a small portion of bioactivities. (**B**) Graph on the right shows the increase in the number of documents of document type ‘DATASET’ in ChEMBL over time (ChEMBL 24–CHEMBL 33).

In addition to bioactivity data extracted from peer-reviewed scientific articles, ChEMBL is also a repository for donated datasets. Indeed, since the first inclusion of deposited datasets into ChEMBL 09 (in 2011), the absolute and relative share of bioactivity data from deposited datasets has grown (Figure 1A). In the latest release (v33), ChEMBL contains a slightly higher number of bioactivity data points from deposited datasets versus bioactivity data extracted from primary literature (∼10.9 Million with document type ‘DATASET’ versus ∼9 Million with document type ‘PUBLICATION’). Data of DOC_TYPE ‘PUBLICATION’ are typically extracted from primary literature, but donated datasets can also include a link to a scientific paper in which the data has been published which would classify these datasets as ‘PUBLICATION’. Data of type ‘DATASET’ include all documents that are deposited/donated to ChEMBL that are not yet published in a scientific journal, as well as data integrated from other public databases (e.g. drug and drug candidate data). For the document types ‘PATENT’ (data from SureChEMBL (21) and BindingDB (22)) and ‘BOOK’, ∼380 thousand and ∼600 bioactivity measurements are available in release 33. The number of unique deposited datasets in ChEMBL has approximately doubled in the past 4 years since the last NAR update (Figure 1B; for more detailed information on newly added datasets since the 2019 NAR update, see section ‘New data sources’).

In total, release 33 of the ChEMBL database (prepared on 31/05/2023) contains information extracted from > 88000 publications and patents (of which 2564 are patents), 420 deposited datasets, and two books for a grand total of >20.3 Million bioactivity measurements and 2.4 Million unique compounds. A comprehensive list of all current datasets incorporated into ChEMBL 33 including some statistics can be found in the latest release notes: https://ftp.ebi.ac.uk/pub/databases/chembl/ChEMBLdb/releases/chembl_33/chembl_33_release_notes.txt

Bioactivities in ChEMBL 33 have been measured in more than 1.6 Million assays and on >17 000 targets of which ∼10 600 are protein targets (including the protein target types ‘SINGLE PROTEIN’, ‘PROTEIN COMPLEX’, ‘PROTEIN FAMILY’, ‘PROTEIN COMPLEX GROUP’, ‘PROTEIN-PROTEIN INTERACTION’, ‘CHIMERIC PROTEIN’, ‘SELECTIVITY GROUP’), and approximately half of these protein targets are human. Non-protein targets include other molecular targets (‘NUCLEIC-ACID’, ‘PROTEIN NUCLEIC-ACID COMPLEX’, ‘SMALL MOLECULE’, ‘MACROMOLECULE’, ‘LIPID’, ‘METAL, ‘OLIGOSACCHARIDE’) with ∼200 different targets, and non-molecular targets (‘CELL-LINE’, ‘TISSUE’, ‘ORGANISM’, ‘SUBCELLULAR’, ‘PHENOTYPE’) amounting to ∼4500 different targets.

**Figure 2.**
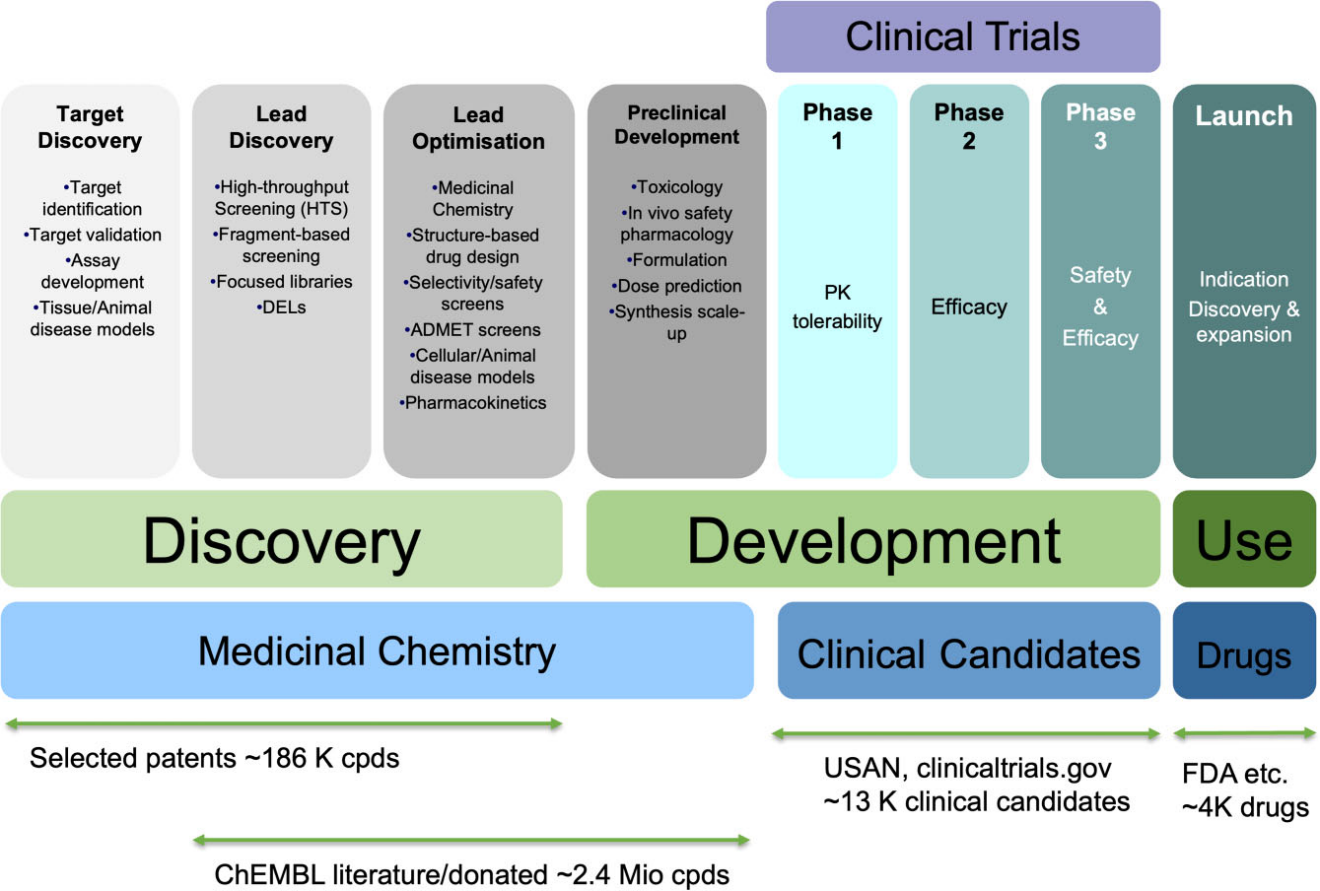
Data in ChEMBL covers all stages of the drug discovery pipeline.

To date, ChEMBL contains bioactivity data covering all stages of the drug discovery process. As depicted in Figure 2, patent data from SureChEMBL and BindingDB covers the earliest phases of target and lead discovery as well as lead optimisation and amounts to ∼186 000 unique compound records. The vast majority of bioactivity data is extracted from the scientific literature and via direct data deposition (∼2.4 million unique compounds) and represents the phases from lead discovery to preclinical development.

### Diversification of bioactivity data types

**Figure 3.**
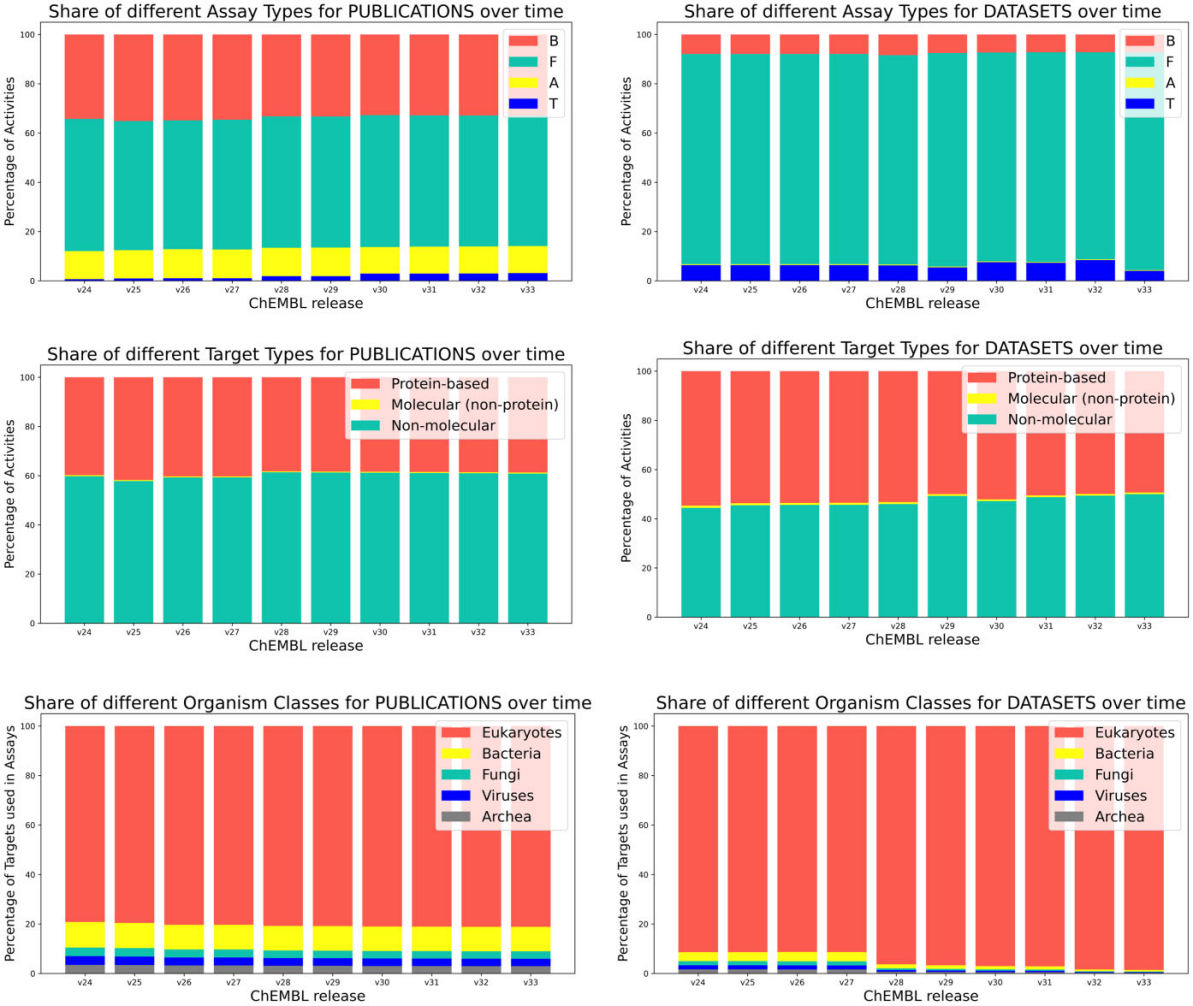
Graphs showing the share of assay types (top panel), target types (central panel), and organism classes (bottom panel) in different ChEMBL releases (v24–v33) for publications (left panel), and for deposited datasets (right panel).

The share of the major assay types in ChEMBL (binding, functional, ADME(T), toxicity assays; for an exact definition please see: https://chembl.gitbook.io/chembl-interface-documentation/frequently-asked-questions/chembl-data-questions) has remained largely constant over the past four years for both data from publications and datasets (Figure 3). For release 30, we re-classified some of the ADMET assays (‘A’) into the more specific category of toxicity assays (‘T’) to allow users to extract these assays separately from other ADME data. As a result, the number of assays flagged as assay type ‘T’ increased six-fold as can be seen in Figure 3. These toxicity assays cover a range of endpoints including hepatotoxicity, selectivity indices (e.g. anti-proliferative activity against cancer cell lines versus cytotoxicity against non-cancer cells) and other therapeutic indices. Comparing data originating from the two major document types ('PUBLICATION’ and ‘DATASET’), significant differences in the share of assay types can be observed: for data from datasets, functional assays are the dominant type with very few ADME assays; for publications, all major assay types are reported to a noticeable degree. Also, the share of high-level target types (protein-based, molecular, non-molecular) has not altered significantly since the last NAR update in 2019 for publications nor datasets. However, comparing publications and datasets, the share of protein-based targets reported in assays from deposited datasets is significantly higher than the share for publications and this trend has remained constant over the past four years (Figure 3). Inspecting the distribution of organism classes for targets reported in assays since release 24, data measured in eukaryotic targets remain the dominant source. However, these dominate to a higher extent for data reported in deposited datasets (esp. from release 28 onwards) vs literature-derived data. For the latter, a substantial fraction of data from bacterial and - to a lesser extent—fungal, viral and archeal targets have been captured with a fairly constant ratio in recent years.

### Diversification of molecule types for drugs

**Figure 4.**
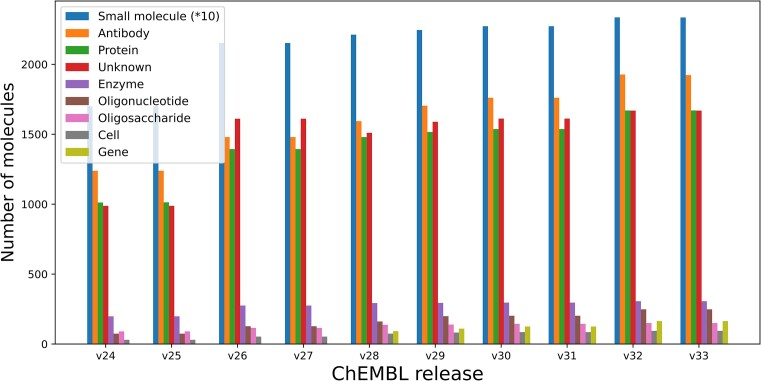
Bar plot showing the numbers of drugs associated with each molecule type across different releases of ChEMBL.

ChEMBL is a key resource for information on approved drugs and drugs progressing through clinical trials (‘clinical candidate drugs’). Investigating how molecule types have evolved for drugs over time (Figure 4), it becomes apparent that while ∼80% of all drugs in ChEMBL are of the type ‘Small molecule’, the latest update of drug data in ChEMBL (for release 32) included a significantly higher number of drugs of type ‘Antibody’ and ‘Protein’. Other categories such as ‘Oligonucleotide and ‘Oligosaccharide’ drugs, and those based on gene therapy (category ‘Gene’ introduced in release 28) have also significantly risen with release 32. Examples of recently added drugs presenting new modalities of treatment are discussed below.

### Diversification of targets and therapeutic modalities

The detailed biological curation process applied to data entering the ChEMBL database has been described previously (23). Since the last update in 2019, there has been substantial growth in number of compounds active against nucleic acid targets (∼93 000 bioactivities). While release 24 included compounds targeting 14 distinct nucleic acid types, release 33 now includes 44 distinct nucleic acid targets. The new targets reflect new modes of action and the increasing use of DNA and RNA in precision medicine. In addition, drugs which target the transcription of DNA, for example promoters, have been introduced in the past 4 years. New targets include pre-mRNAs, and an increasing number of microRNA targets (∼65 000 bioactivities). There is also an increase in structured nucleic acids, for example tRNAs, G-quadruplexes and a riboswitch.

An area in which growth can be expected is gene therapies, examples include Zolgensma (CHEMBL4297240) and Sipuleucel-T (CHEMBL1237024). The FDA currently reports 29 approved gene therapies, with more awaiting approval or in clinical trials; 12 of these are included in the current ChEMBL release. Another notable shift in modalities since release 24 is the increase in proteolysis targeting chimeras (PROTACs). PROTACs are heterobifunctional small molecules that catalyse the degradation of a target protein. They contain two distinct moieties: one binds a disease-relevant drug target (the protein of interest) and the other one an E3 ubiquitin ligase binding moiety. E3 ubiquitin ligases are part of the ubiquitinylation complex that tags proteins with ubiquitin and identifies them for degradation by the cellular degradation machinery (proteasome). Placing pathogenic proteins in proximity to ubiquitin ligases, using PROTACs, can promote degradation of disease-causing proteins without a need for high affinity binding to a protein binding site (24).

The number of bioactivity data points from assays testing PROTACs has increased significantly, from 85 to 6942 bioactivities. Although there are currently no FDA-approved PROTACs, over twenty compounds are in clinical trials (e.g. ARV-471, ARV-110). As PROTACS progress towards approval, more comprehensive data on their indications and mechanisms will appear in ChEMBL. Human cereblon and von Hippel ligase (VHL) are two of the most common E3 ubiquitin ligases used in PROTACs; older drugs that function as Cereblon-binding PROTACs (THALIDOMIDE, POMALIDOMIDE, LENALIDOMIDE, IBERDOMIDE) are already curated with their Cereblon drug target.

### New and updated data sources

Since the 2019 description of the database, several new data sources have been incorporated into ChEMBL, as described below.

#### Patent bioactivity data (source ID 38)

Since release 24, we have continued our patent curation efforts. We have focused on identifying patents with bioactivity data for small molecules against understudied targets, in collaboration with the Illuminating the Druggable Genome project (25). The workflow for identifying such patents has been published recently (26). As a result, a dataset of 381 new patents extracted from the SureChEMBL database (21) has been added to ChEMBL, containing 99948 bioactivity values against 1322 targets. For 154 of these targets, patents are currently the only source of bioactivity data.

#### SARS-CoV-2 screening data 2020-21 (source ID 52)

The SARS-CoV-2 pandemic was an exceptional event that prompted research into drugs with activity against the causative pathogen, SARS-CoV-2. To contribute towards this effort, release 27 was a special release that focused on the integration of drug repurposing data, targeting SARS-CoV-2 infection/replication in cell-based assays, into ChEMBL (27).

#### Donated Chemical Probes—SGC Frankfurt (source ID 54) & EUbOPEN Chemogenomics library (including literature data) (source ID 55 and 65)

The EUbOPEN consortium (https://www.eubopen.org/) is an Innovative Medicines Initiative 2 (IMI 2) funded, 5-year public-private partnership with the aim to assemble an open access chemogenomic library comprising about 5000 well annotated compounds covering roughly one thousand different proteins and to design and synthesise at least one hundred high-quality, open-access chemical probes. Other objectives include the establishment of an infrastructure to generate and characterise these probes and chemogenomic compounds including the public dissemination of project results. The ChEMBL database was chosen for the long-term public storage of project results and has been augmented by a bespoke data platform called the EUbOPEN Gateway (https://gateway.eubopen.org/). As part of this initiative, bioactivity data for 206 chemical probes measured on 1435 distinct targets (70.8 thousand measurements) have been incorporated into ChEMBL from the Donated Chemical Probes resource (https://www.thesgc.org/donated-chemical-probes) from release 28 onwards (source ID 54). In addition, a chemogenomic library has been assembled since ChEMBL release 29 with currently 933 compounds measured on a total of 180 targets (corresponding to ∼400 000 activities). An additional ∼2800 bioactivity measurements have been extracted from the primary literature by the EUbOPEN consortium to complement the Chemogenomic library and have been added to ChEMBL by creating a new source (Literature data from EUbOPEN Chemogenomic Library, source ID 65). References to primary literature are indicated in the ACTIVITY_PROPERTIES table (TEXT_VALUE AND STANDARD_TEXT_VALUE fields).

#### Resolute—research empowerment on solute carriers (source ID 58)

This dataset was added to release 33 and comprises 96 bioactivities measured in 34 assays on 32 solute carrier (SLC) targets from the IMI-RESOLUTE project. RESOLUTE (https://re-solute.eu) is an EU-funded consortium working on the SLC gene family in a public-private partnership. The consortium also develops new transport assays for selected SLCs.

#### Drug and clinical candidate drug data

For release 33, there were 11 544 compounds with USAN or INN applications; 8415 compounds that were recorded to have reached at least Early Phase I clinical trials, and 2993 approved drugs (using counts of parent compounds rather than individual salt forms). In addition (for parent drugs or clinical candidate drugs), 7590 compounds have at least one indication annotated; 5392 compounds have at least one mechanism annotated; 592 have at least one black box warning annotated; and 202 have been withdrawn. Compounds with INN applications were added as a new clinical candidate drug source for release 32 (source ID 63).

#### Prodrugs

Prodrugs are drugs that are inactive until metabolised *in vivo* to active metabolites. They may have improved pharmacodynamic and pharmacokinetic properties or may be activated in specific microenvironments, such as hypoxic tumours, and therefore can access disease-relevant tissues with fewer off-target effects. In addition to the prodrug flag, the pharmacologically active ingredient of a prodrug (source ID 53) has been recorded within the molecule hierarchy as the ‘active_molregno’ since release 28. However, only one active ingredient for each prodrug is allowed in ChEMBL, so intermediate active ingredients are not stored. The number of curated prodrug families has nearly doubled to 400 (from 223 in release 24) improving the annotation of drugs by flagging those structures with masked activity. Prodrug curation is ongoing and recent efforts to further enhance this carefully curated subset of drugs is likely to reveal increasingly complex drug mechanisms, targeting and delivery features.

#### CO-ADD antimicrobial screening data (source ID 40)

As part of the not-for-profit initiative CO-ADD (Open-access antimicrobial screening program) led by the University of Queensland (https://www.co-add.org/) to combat drug-resistant infections, 31 additional datasets (almost 100 thousand new bioactivity measurements) have been deposited in the ChEMBL database since release 24.

#### Kuster lab chemical proteomics drug profiling (source ID 48)

∼70 500 bioactivity measurements for a set of 243 clinically evaluated kinase drugs on 320 targets were included in release 25 (28).

#### HESi (source ID 49)

ADME type assays were reported in release 26 for a dataset with stem cell-derived cardiomyocytes (PSC-CMs) to evaluate use in an *in vitro* proarrhythmia model. Data includes electrophysiological responses to 28 drugs linked to low, intermediate, and high torsades de pointes (TdP) risk categories using multiple cell lines and standardised protocols.

#### Winzeler lab Plasmodium screening data (source ID 51)

For release 28 we included a large screening dataset (∼400 000 activities for 78 000 compounds) which was measured against Plasmodium e.g. for their ability to inhibit liver-stage development of luciferase-expressing Plasmodium spp. Parasites (29,30).

### Global data usage

The ChEMBL database continues to be a globally used resource with a monthly average of ∼53 000 visits/sessions, ∼25 000 visitors/users and ∼531 000 page views. Investigating demographic details of ChEMBL usage within the last three months (mid May until mid August 2023), the USA is ranked top with ∼14 000 users, followed by China (∼10 000), Serbia and India with ∼7000 users, the UK (∼3000 users), Japan, Germany, Finland, South Korea and Italy with ∼2000 users, respectively.

**Figure 5.**
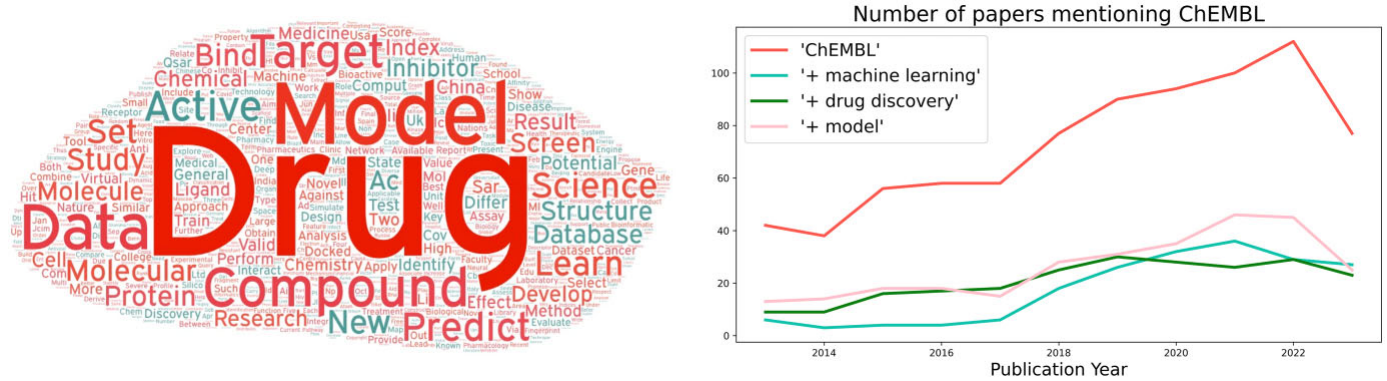
Left: word cloud of all abstracts of papers in PubChem mentioning ChEMBL within the last 5 years; Right: time trends of papers mentioning ChEMBL together with other drug discovery related terms within the past 10 years.

Next, we investigated the time trends of articles listed in PubMed, which were published in the past 10 years and mentioned ‘ChEMBL’ in their title and/or abstract, as well as articles mentioning the terms ‘ChEMBL’ AND ‘machine learning’, ‘ChEMBL’ AND ‘drug discovery’, as well as ‘ChEMBL’ AND ‘model’. As seen from Figure 5, over the past ten years, articles in PubMed which mention these terms (in combination) steadily increased, underpinning ChEMBL’s usefulness in those thematic areas. Further the abstracts of all articles mentioning ‘ChEMBL’ in title or abstract between 2019 and 2023 were used as the basis to generate a thematic word cloud (with https://wordart.com/). Certain ubiquitous words have been removed for the visualisation (such as ‘DOI’, ‘PMID’, ‘ChEMBL’, ‘author’, ‘department’). The resulting word cloud confirms ChEMBL’s central role in drug discovery and predictive modelling.

## New developments

### Chemical structure standardisation

ChEMBL contains ∼2.4 million unique chemical structures which, as part of the ChEMBL curation process, must be standardised. In collaboration with Dr Greg Landrum, we developed a new curation pipeline to standardise chemical structures in preparation for molecular modelling applications, described in Bento *et al.* (31). The curation pipeline is publicly available via GitHub (https://github.com/chembl/ChEMBL_Structure_Pipeline) as both conda and PyPl repositories, to facilitate ready accessibility for users. The new curation pipeline consists of three separate components. The first step is a Checker which validates the chemical structures and identifies any (serious) problems before a compound is added to the ChEMBL database. If there are any issues with the structure, the compound is given a penalty score. The penalty score ranges from 2 (low priority and usually due to unavoidable issues like rearranging charges) up to 6 (compound loaded into ChEMBL but without the structure). The highest penalty score is 7 which is considered a fatal error and the compound will not be loaded into ChEMBL.

The next step of the curation pipeline is the Standardizer component. During this process the chemical structure is corrected according to a set of predefined rules based largely on the FDA/IUPAC guidelines (https://www.fda.gov/industry/fda-data-standards-advisory-board/fdas-global-substance-registration-system) (32).

The third and final step is the GetParent function in which the parent molecule is created based on a set of rules and defined lists of salts and solvents. The list of salts is based on the USAN Council's list of pharmacological salts (https://www.ama-assn.org/system/files/2019-04/radicals-and-anions-list.pdf). Both salts and solvent files are available in the GitHub repository and currently contain 163 salts and 9 solvents (https://github.com/chembl/ChEMBL_Structure_Pipeline/tree/master/chembl_structure_pipeline/data).

### Protein variants

Protein variation may be associated with disease or drug resistance. In addition, variation can impact the ADMET properties of drugs. Knowledge of protein variation can inform the development of selective drugs such as the oncology compound Encorafenib that targets B-raf mutants such as V600E, the chaperone Migalastat that stabilises protein variants associated with Fabry disease and the third-generation oncology drug Ponatinib that overcomes the ABL T315I resistance mutation. The pharmacokinetic properties of Rosuvastatin are impacted by the SLC01B1 rs4149056 variant and this drug is contraindicated in patients with this genotype (https://www.fda.gov/drugs/science-and-research-drugs/table-pharmacogenomic-biomarkers-drug-labeling). Target variation was first included in ChEMBL 22 and is recorded in the VARIANT_SEQUENCES table. A drive to curate legacy variant data was undertaken for release 28. Overall, 2443 curated variants are included in ChEMBL and 13 323 assays (0.83%) assess variant proteins. There is potential to further classify the mutation type (natural, acquired, resistance) and function (e.g. activating, resistance, alanine scan, loss-of function, crystallisation) in future releases.

### Curation of drug and clinical candidate drug data

Curation of data for approved drugs, and clinical candidate drugs, has formed an integral part of the core offering of the ChEMBL database since its inception. The multiple processes to extract and curate new drug and clinical candidate data, and integrate it with existing data, involve much manual and semi-automated curation. We aim to provide the highest quality data and so many checks are performed as part of these processes. Where there are differences between various input data sources for an individual drug or clinical candidate drug, in-depth discussion within our curation team may be needed before a conclusion can be reached. The detailed nature of these curation processes means that the drug and clinical candidate data is updated approximately every other ChEMBL release with the next drug and clinical candidate data update planned for ChEMBL 34.

Approved drug and clinical candidate drug data are extracted from a variety of sources as described in the 2019 NAR update. For release 32, the WHO International Nonproprietary Names proposed list of compounds (INN (1)) was introduced, and the categories for maximum phase of development reached for the compound across all indications (‘MAX_PHASE’) were revised (4 = Approved, 3 = Phase 3 Clinical Trials, 2 = Phase 2 Clinical Trials, 1 = Phase 1 Clinical Trials, 0.5 = Early Phase 1 Clinical Trials (https://prsinfo.clinicaltrials.gov/definitions.html), −1 = Clinical Phase unknown for drug or clinical candidate drug i.e. where ChEMBL cannot assign a clinical phase). For example, unknown status could be assigned if the compound is not regulated for human medicine (e.g. a veterinary drug) or is an old compound that progressed through clinical trials prior to the first release of the ClinicalTrials.gov resource). More detailed information can be found here: https://chembl.gitbook.io/chembl-interface-documentation/frequently-asked-questions/drug-and-compound-questions#what-is-max-phase.

### *In vivo* assay classification

ChEMBL contains a wide range of pharmacological data at varying scales of biological complexity, including around 772000 functional assays that investigate the biological effect of individual compounds in complex cell-, tissue-, organ-based or whole animal models. Typically, this data is under-utilised by our user community and to improve its accessibility, an *in vivo* assay classification was first introduced for release 24 and has been curated for each subsequent release. Currently, there are 120 328 assays with an *in vivo* classification; full details are available in Hunter et al. (33).

### Curation of PK/PD data

To improve the usage of pharmacokinetic data stored in the database, we have reviewed the units in which these records are stored to deliver greater consistency and standardisation. Where possible, unit conversions are performed, as this delivers a more homogeneous set of data for users. For release 33, we focused on AUC and Cmax data. As a result, 91% of the AUC data are now expressed in ‘ng h ml^–1^’ against 62% previously, and 97% of the Cmax data are none expressed in ‘nM’ against 37% previously.

Work is on-going to further improve the homogeneity and to deliver more comprehensible parameters (time, dose, route of administration) associated with these endpoints. A similar approach could also be extended to other pharmacokinetic endpoints.

### New target prediction

**Figure 6.**
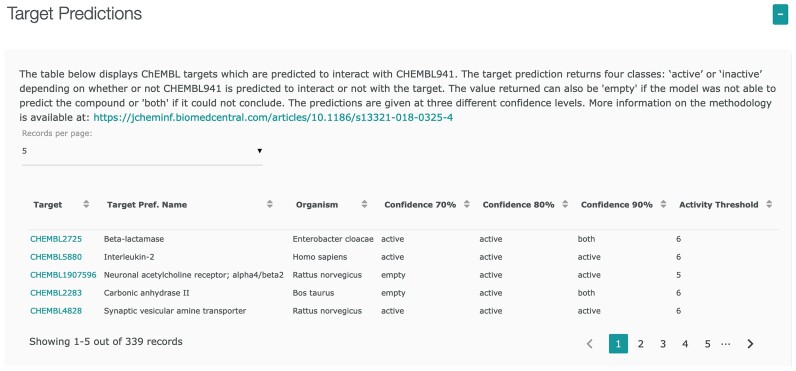
Target predictions based on conformal prediction models available via the ChEMBL web interface. The example shows predictions for imatinib (CHEMBL941).

Since release 26, we have used a new approach for the target prediction module introduced with release 18. We switched from a single multi-class multinomial Naive Bayes model to a set of mono-task classification Random Forest models making use of the conformal prediction framework (10). The predictions can be accessed through the ChEMBL web interface where every compound gets a table showing a predefined list of predicted protein targets with the associated confidence (see Figure 6 for an example).

### Harmonisation of journal name abbreviations

Journal names have been harmonised and standardised according to the National Library of Medicine (NLM) standards for release 32.

### Updates to UniChem

ChEMBL uses the UniChem (https://www.ebi.ac.uk/unichem/) (34) database to provide cross-references from a given Compound report card to other chemical databases. Since the last published update to UniChem in 2014, it has grown from over 65 million unique structures to >178 million. We have since added 21 new sources, almost doubling the number of referenced databases. The UniChem interface has been updated to a v2.0 version written in Vue 2 and Nuxt 2. In addition to the previous search methods (InChI, InChI Key or internal DB ID), users can now search by drawing the molecule in Marvin JS.

The UniChem web services now implement current REST standards such as JSON payloads for the request and response, allowing a more intuitive experience for the users. The way the services are called, and its response has been greatly simplified but retains the same functionality. Full information on the new website and web services can be found here (https://chembl.gitbook.io/unichem/whats-new).

The UniChem documentation can be found at https://chembl.gitbook.io/unichem.

## New features

### Natural product flag and natural product likeness score

In recent years, we have seen a revival of interest in drug discovery inspired by natural products (35). To facilitate investigation in this research area, we flag compounds produced by a living organism as natural products (NPs) and from release 32 onwards we also provide a score to estimate the NP likeness of a small molecule. The Natural Product (NP) flag was revised for release 33 to use a newer implementation based on mappings to COCONUT, an open-source collection of NPs (36). For the structure mapping, stereochemical information in the ChEMBL compound structures is ignored as compound structures in COCONUT did not include stereochemical information when the mapping was performed. The NP flag (0/1) is available from the MOLECULE_DICTIONARY table (NATURAL_PRODUCT). Currently, ∼64 000 molecules in ChEMBL release 33 are flagged as NPs. One potential limitation of the current mapping approach is that NP salts of parent compounds that are themselves NPs are currently not flagged as such.

The new NP likeness score is based on the method published by Ertl *et al.* (37) and has been calculated based on an open-source implementation in RDKit (https://github.com/rdkit/rdkit/tree/master/Contrib/NP_Score). It gives a value between –5.0 and 5.0 and is available from the COMPOUND_PROPERTIES table (NP_LIKENESS_SCORE).

### Chemical probes flag

Chemical probes are highly selective modulators of drug discovery relevant targets, which can serve as valuable tools to help decipher target biology (38). To allow easier tracking of a probe's publication history, we flag chemical probes within a new field in the MOLECULE_DICTIONARY named CHEMICAL_PROBE. This indicates whether the compound is a chemical probe as defined by chemicalprobes.org (1 = yes, 0 = default value). The dataset of chemical probes was retrieved from the chemicalprobes.org website and filtered for probes that were assigned an *In Vivo* Rating or In Cell Rating of 3 stars or more. The annotations will be updated with every new ChEMBL release and other sources of information for defining a chemical probe may be added in the future. ChEMBL release 33 currently includes 388 molecules flagged as chemical probes.

### Drug warning information

Drug safety data continues to be curated for each ChEMBL release. For release 32, the set of approved drugs that have subsequently been withdrawn from the market for toxicity reasons (‘withdrawn drugs’) was fully reviewed and, to assist the manual curation process, our rules were updated, clarified and formally written up (see https://chembl.blogspot.com/2023/03/drug-warning-update-withdrawn-drugs-and.html). Each withdrawn drug includes a citation to a regulatory document or similar; the specific (granular) withdrawn reason is mapped to EFO (e.g. the phrase 'cardiac arrhythmia’); and a high-level toxicity class is assigned as the warning class and mapped to EFO (e.g. the phrase ‘cardiotoxicity’). FDA drugs that carry a black box warning for a severe or life-threatening side effect(s) continue to be curated (39). Since release 32, the high-level toxicity class (e.g. cardiotoxicity) is also mapped to EFO.

### Time stamping documents

For release 33, a new table has been added (CHEMBL_RELEASE) which provides the creation date for every ChEMBL release. In addition, a new field has been added to the DOCS table termed CHEMBL_RELEASE_ID which links to the new CHEMBL_RELEASE table. Thus, for every document in ChEMBL the version of the database when the document was added, and its creation date, is now available. This new feature facilitates any study that require the time dimension, or a time split of datasets, or when analysing when a specific dataset has been added.

### Action type

The action types of drugs and clinical candidate drugs continues to be curated and provides information on the mode of action of a drug or drug candidate, e.g. inhibitor, blocker, inverse agonist). A total of 32 different action types and their definitions are captured in the ACTION_TYPE table. From release 33 onwards, we have also provided action type information for a selection of preclinical compounds with bioactivity data. This information has been made available via the ACTIVITIES table (field ACTION_TYPE). The recorded ACTION_TYPE must match one of the names in the ACTION_TYPE table. This field was populated with mode of action information that had previously been recorded as metadata in the ASSAY_PARAMETERS and ACTIVITY_PROPERTIES tables. In addition, since release 30 the action type has been manually annotated by the ChEMBL data extractors (but not made publicly available until release 33). The initial subset of ∼270 K action types for curated activities are being released as a test set and we encourage feedback. As the rules are being refined over time and atypical cases identified, a small number of annotations may change over the coming releases.

## Data deposition

### Improved data deposition protocol and documentation

The basic loader documentation has been redesigned into a public-facing open manual that provides the file standards for depositing data into ChEMBL and guides a potential depositor through the process. This guide can be found at https://chembl.gitbook.io/chembl-data-deposition-guide/. It starts out with the description of a minimal submission and provides an example template dataset which the depositor can use as a base for their own data structure. It then covers advanced deposition options like supplementary data, linking multiple data types using the Test Occasion ID, and how one might use identifiers to link together groups of assays from the same plate-based experiment. The goal here is to ensure that depositors will submit data in consistent ways, and to reduce the amount of time spent on error-checking user data. The documentation also covers common errors and issues in deposited data, and how to fix these.

Previously, data deposition and loading were informally organised, with releases after major submissions. We have moved to a process where we formally announce that ChEMBL is open for submissions and have a clear deadline for depositors to submit by. This has allowed for a faster turn-around of datasets, and enabled ChEMBL to move to a defined release cycle.

We have worked with some of our repeat depositors (e.g. EUbOPEN) to produce a selection of training and admin resources to aid new depositors. E.g.:

- A one-page summary of the deposition process is available as Supplementary information (Supplementary File S1).
- A checklist that allows users to confirm that their input files have all the necessary data and formatting to be valid for loading available (Supplementary File S2).
- A short video explaining the ChEMBL deposition process: https://embl-ebi.cloud.panopto.eu/Panopto/Pages/Viewer.aspx?id=4b4d09da-ce29-4b72-b649-b0750115cad7

## Improved documentation and new training materials

### New FAQ’s

As well as further improvements to the database, we have continued to develop the documentation which accompanies ChEMBL. Our guide to using the web interface for ChEMBL includes links to additional web services and a comprehensive FAQ section. However, the documentation has recently been updated and expanded, to reflect current practices and recurrent themes in response to questions that we have received. Extensively reorganised into sections which better reflect the use cases of the database, with additional answers to frequently asked questions, the FAQs contain information on our data provenance, curation practices at ChEMBL, data standards and technical help. The ChEMBL Web Interface Documentation can be found at https://chembl.gitbook.io/chembl-interface-documentation/.

### Training material

ChEMBL offers freely available and tailored training to both industry and academic users. Sessions can be arranged upon request in both virtual and in-person formats (https://www.ebi.ac.uk/training/events/chembl-and-surechembl-bioactive-molecules-targets-and-patent-resources/). On-demand training materials are also available through the EBI (https://www.ebi.ac.uk/training/on-demand) and provide an overview of the ChEMBL data content, curation and standardisation process with an emphasis on worked examples. Materials are periodically updated alongside dedicated training sessions following major changes to the database and/or access methods. Following the release of the new ChEMBL interface in early 2019, guidance on how to navigate the new interface was provided ('Exploring ChEMBL Data with the new ChEMBL Interface’, https://www.ebi.ac.uk/training/events/exploring-chembl-data-new-chembl-interface-0/). In early 2021, a new webinar providing an overview of ChEMBL alongside a worked drug discovery example was given (https://www.ebi.ac.uk/training/events/guide-explore-drug-compounds-and-their-biological-targets-using-chembl/). This also offered an opportunity to gain feedback from users which prompted an review of our natural product offering within ChEMBL and the potential annotation of the mode of action of preclinical compounds (see above). In late 2021, an update to the ChEMBL and UniChem web services training was provided and covered new features and functions alongside a worked example in ChEMBL (https://www.ebi.ac.uk/training/events/guide-accessing-chembl-and-unichem-through-api/). With a goal to make our training materials FAIRer, all presentation materials and Jupyter notebooks (for worked examples) are available for download through the EBI and additional platforms (such as YouTube). Guidance on ChEMBL is also provided through our dedicated Helpdesk (chembl-help@ebi.ac.uk). In addition to training, ChEMBL is also actively involved in a range of internal and external public engagement activities and welcomes enquiries through the main ChEMBL Helpdesk.

## Data access

### The ChEMBL Web Interface

In 2019, the original web interface was replaced by a redesigned application, leading to the discontinuation of the old version. Links to the old version now direct users to the new interface. The current web interface has been improved since its initial release in 2019, with additional enhancements detailed in subsequent paragraphs. Some functionalities initially created for the web interface have been transformed into standalone applications that can be reused across team projects.

### Backend enhancements

#### Deployment in Kubernetes cluster

All the services involved in ChEMBL’s data storage and serving services have been migrated to a Kubernetes (https://kubernetes.io/) cluster, a system that automates application deployment and management. The web interface and web services transitioned from dedicated virtual machines to this cluster, resulting in improved scalability and reliability for the interface.

#### In-memory cache

In the Kubernetes cluster, a Memcached cache (memcached.org) system boosts ChEMBL interface performance. This is ideal for retaining precomputed data with limited updates, speeding up data access, including visualisations. For repeated visualisation access, data is computed and then stored in the cache, reducing redundant calculations. This aligns well with ChEMBL’s data pattern, which remains static until the next data release.

#### Delayed jobs system

The web interface provides functionalities that involve time-consuming tasks, such as dynamic CSV file generation, through an asynchronous job system that utilises an LSF cluster for execution. This sub-system has also been repurposed for other projects within the ChEMBL Team, such as our malaria prediction tool MAIP (40).

### Structure search enhancements

#### Similarity search

The FPSim2 Python package (10.5281/zenodo.7781320) was developed and open-sourced (https://github.com/chembl/FPSim2) with the intention of replacing RDKit's PostgreSQL cartridge within the ChEMBL service. FPSim2 is a fast, in-memory Python/C++ specialised tool designed for executing similarity searches based on molecular fingerprints. Molecules are characterised as Morgan fingerprints with a radius of 2 and 2048 bits of length.

#### Substructure search

RDKit's PostgreSQL cartridge was replaced with RDKit's SubstructLibrary to enhance searches performance.

#### Connectivity search

The connectivity search was enhanced to operate within the existing ChEMBL Elasticsearch instance, executing an exact text match of the first block of the InChI key.

### Frontend enhancements

The ChEMBL web interface undergoes continual improvement, with new features and enhancements being added on a regular basis. These, and other announcements, are typically reported in the ChEMBL blog (http://chembl.blogspot.com/).

#### Modern javascript frameworks

The 2019 version used Django (www.djangoproject.com) and Backbone.js (backbonejs.org). Currently, sections with Vue.js (vuejs.org) and Nuxt.js (nuxt.com) are being incrementally added, taking advantage of their modern features like reactivity and component reusability. These frameworks are also being employed in other ChEMBL projects for easier maintenance and sustainability.

#### New search type

**Figure 7.**
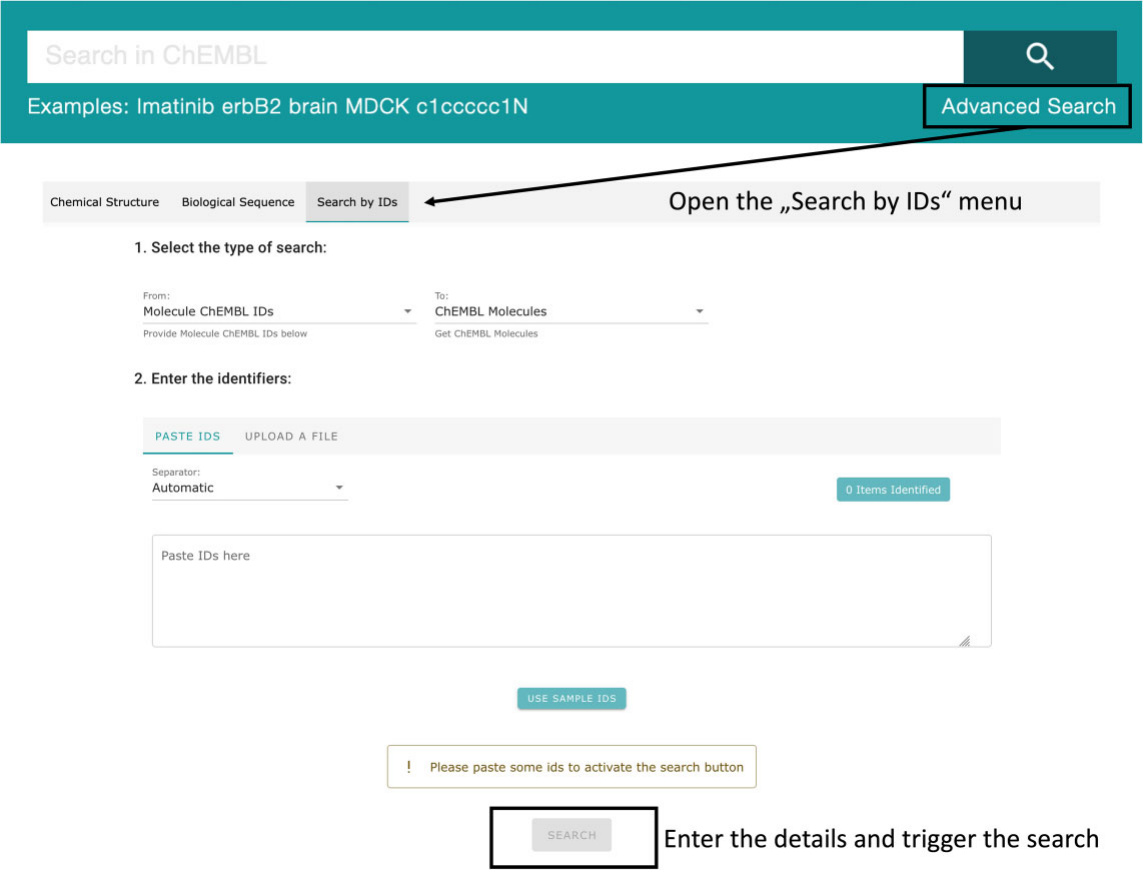
Image of the Search by IDs dedicated menu.

A new type of search has been introduced. The ‘Search by IDs’ feature (Figure 7) for direct retrieval of ChEMBL items from user-input IDs is accessible under ‘Advanced Search’. This specific search offers tailored results with status of the searched IDs (active, obsolete, non-matching).

### FTP downloads

FPSim2 fingerprint database file, as detailed in the Structure search enhancements section, has been incorporated into the ChEMBL downloads repository since the release of ChEMBL 32.

Oracle database dumps were excluded from the primary ChEMBL downloads repository starting from ChEMBL 29. The Oracle 19c version of ChEMBL remains accessible upon user solicitation until ChEMBL 34, at which point the support will be concluded by the team.

## Summary

The ChEMBL database has become a repository for multiple data sources and data types over the past ∼14 years, providing drug discovery relevant information for chemical biology and all stages of drug discovery. ChEMBL is also a valuable resource for machine learning applications, given its large size and extensive coverage of chemical and target space. Another key attribute of ChEMBL is the high quality of the data and the wealth of annotations it contains, due to its still largely manual curation and annotation process.

In this update, we have described the types of data that ChEMBL currently holds, and described some new features that, e.g. improve searches for specific data types and time ranges. Other enhancements described include an improved, open-source chemical structure standardisation pipeline and the addition of target predictions. ChEMBL’s increasing diversity of data types and growing number of new depositors, was a main motivator to also start facilitating the deposition process for data into ChEMBL. Mainly by simplifying the deposition guide, providing short videos and a checklist for data depositors. In the future, we plan to work even closer with the community to help the continuous data flow into ChEMBL.

## Supplementary Material

gkad1004_Supplemental_Files

## Data Availability

The ChEMBL database is made available under a Creative Commons Attribution-ShareAlike 3.0 Unported license (http://creativecommons.org/licenses/by-sa/3.0).

## Notes

Present address: Anna Gaulton and A. Patrícia Bento, Exscientia, The Schrödinger Building, Oxford Science Park, Oxford OX4 4GE, UK.
