# Supplementary material for: The ChEMBL Database in 2023: a drug discovery platform spanning multiple bioactivity data types and time periods
Source: Nucleic Acids Res. 2023 Nov 2;52(D1):D1180–92. doi: 10.1093/nar/gkad1004 (PMC10767899; doi:10.1093/nar/gkad1004)
Supplement: gkad1004_Supplemental_Files [file gkad1004_supplemental_files.zip › Supplementary_File_S1.pdf]

Introduction to the ChEMBL deposition process

Preparation

If you wish to be informed when we are accepting depositions for the upcoming release of ChEMBL, please sign up to the mailing list here.

Once deposited, your data will be stored and made available in the standard ChEMBL format. Our database cannot create a custom data structure for each deposition.
As such, we strongly recommend that you are familiar with ChEMBL before you submit data.
If you have not used ChEMBL much, we have a half-hour introductory course here.

Please contact chembl-deposition@ebi.ac.uk and let us know:

- Your name and institution.
- The sort of data you are intending to deposit to ChEMBL.
- A suggestion for a time slot for a follow-up call to discuss your data.

If you can put together a subset of example data in the **ChEMBL deposition format**, please attach it to the email. Otherwise, please provide us with an example set of your data (in xls or csv/tsc format), so we can have a look at it.
A summary of the load process you can find here, which links to our further documentation.

Formatting

You can see an example of some valid data here. It contains some fields that are optional.
There is a list of accepted deposition files here.

Producing the ChEMBL-formatted files

Producing files suitable for a ChEMBL deposition may look complicated. However, it’s straightforward once you’re familiar with the table names and fields, and how these should be populated with your data.
We can arrange an initial video call where we’re happy to walk you through the process.
We also have a short video guide to producing these files, using an example dataset, as well as a checklist you can use to check for most common data issues.

Deposition

Once we get back to you, you can then send a set of ChEMBL-formatted deposition files and we will test them. If these load to the test database successfully, we will reply to you and inform you of this.

You can find an overview of the full deposition process as a flowchart here.
