# Supplementary material for: The ChEMBL Database in 2023: a drug discovery platform spanning multiple bioactivity data types and time periods
Source: Nucleic Acids Res. 2023 Nov 2;52(D1):D1180–92. doi: 10.1093/nar/gkad1004 (PMC10767899; doi:10.1093/nar/gkad1004)
Supplement: gkad1004_Supplemental_Files [file gkad1004_supplemental_files.zip › Supplementary_File_S2.pdf]

ChEMBL Deposition quick checklist

This checklist has been designed to catch the most common errors in deposited ChEMBL data. If you check your data against this list, it will substantially reduce the risk that your data will require corrections.

Depositor-Defined Identifiers:
- Do your RIDX, AIDX and CIDXs all give a meaningful unique identifier to each reference compound and assay in their datasets?
- Is every RIDX a unique, non-redundant reference to a single dataset or publication?
- Does every CIDX refer to a unique compound?
- Does every AIDX refer to a unique assay?

ASSAY:
- Does every Assay have an RIDX that links to a REFERENCE in this dataset?
- Does every assay have a description, assay_organism and assay_tax_ID?
- Are variants, isoforms and other mutations captured within the assay description?
- Are cell-lines, tissues and subcellular fractions captured in the corresponding assay_cell_type, assay_tissue and assay_subcellular_fraction columns?
- For functional cell-based assays, has the target protein been recorded as well as the cell-line?

ASSAY PARAMETERS:
- Are there assay parameters that should be captured as part of the assays (e.g. cell-lines, concentrations, tissues can be captured as part of the assays table)?.

ACTIVITY:
- Does every CRIDX match a valid RIDX in this dataset?
- Does every ACTIVITY have a VALUE or TEXT VALUE.
- Does every VALUE have a RELATION and UNIT field?
- Does every TEXT_VALUE have a blank RELATION field?
- Are any TEXT_VALUES actually a VALUE plus a RELATION, and therefore should be in these fields?

ACTIVITY_PROPERTIES:
- Do the ACT_IDs in ACTIVITY match to the ACT_IDs for the relevant ACTIVITY_PROPERTIES and vice versa?
- Does every ACTIVITY have a VALUE or TEXT VALUE.
- Does every VALUE have a RELATION and UNIT field?
- Does every TEXT_VALUE have a blank RELATION field?
- Are any TEXT_VALUES actually a VALUE plus a RELATION, and therefore should be in these fields?

REFERENCE:
- Does every Reference have a TITLE, AUTHORS and ABSTRACT?
- Do all DATASETS have a title, authors and an abstract that provides an easy to understand explanation for database users?

COMPOUND_CTAB:

- Is every CTAB valid? You can check using the 'check' endpoint of the ChEMBL APIs here.
- Is every CIDX in the SDFile a CIDX that is in the COMPOUND_RECORDS file?

COMPOUND_RECORDS:

- Have both a compound_name and a compound_key (this is often a depositor identifier) been supplied?
